# Brief Mindfulness Meditation Improves Mental State Attribution and Empathizing

**DOI:** 10.1371/journal.pone.0110510

**Published:** 2014-10-17

**Authors:** Lucy B. G. Tan, Barbara C. Y. Lo, C. Neil Macrae

**Affiliations:** 1 School of Medicine, Psychiatry Discipline, University of Queensland, Brisbane, Australia; 2 Department of Psychology, University of Hong Kong, Hong Kong, China; 3 School of Psychology, University of Aberdeen, Scotland, United Kingdom; The University of Chicago, United States of America

## Abstract

The ability to infer and understand the mental states of others (i.e., Theory of Mind) is a cornerstone of human interaction. While considerable efforts have focused on explicating when, why and for whom this fundamental psychological ability can go awry, considerably less is known about factors that may enhance theory of mind. Accordingly, the current study explored the possibility that mindfulness-based meditation may improve people’s mindreading skills. Following a 5-minute mindfulness induction, participants with no prior meditation experience completed tests that assessed mindreading and empathic understanding. The results revealed that brief mindfulness meditation enhanced both mental state attribution and empathic concern, compared to participants in the control group. These findings suggest that mindfulness may be a powerful technique for facilitating core aspects of social-cognitive functioning.

## Introduction

*“It is wisdom to know others; it is enlightenment to know one’s self.”*

*Lao-Tzu, 6^th^ century BC*

Interpersonal success rests on the mind’s remarkable ability to decode and comprehend other people’s mental states. In a challenging social landscape, mindreading is the conduit through which relationships are forged, cultivated, and sustained [1], [2]. As Lao-Tzu’s quote indicates and an extensive literature has demonstrated, both admirable (e.g., empathy, compassion) and unsavory (e.g., deception, manipulation) aspects of the human condition rest squarely on the ability to read minds [3]. Indeed the utility of this skill – commonly referred to as Theory of Mind (ToM) – is most apparent when one considers the plight of individuals for whom mindreading is a decidedly problematic activity (e.g., autism spectrum disorder), a topic that has dominated psychological writings for decades [4]–[6]. But what of the converse situation, rather than focusing on impairments in mindreading, is it possible to identify factors (e.g., interventions) that may enhance ToM?

Intriguingly, preliminary investigations have revealed that mindreading can be improved under certain circumstances. Nasal administrations of oxytocin, reading literary fiction, and extended compassion-based training have all been shown to enhance ToM [7]–[9]. Extending this line of inquiry, here we considered whether a brief period of mindfulness-based meditation would similarly improve mindreading performance. In recent years, mindfulness interventions have been shown to remediate a range of clinical problems (e.g., depression, anxiety, stress) and to impact core aspects of social cognition (e.g., metacognition, self-referential thought, see [10]). Emphasizing the non-judgmental appraisal of present-moment thinking [11], [12], even brief episodes of mindfulness meditation exert profound effects on brain and behavior, effects that we suspect may extend to people’s mindreading skills.

Several strands of research highlight important linkages between mindfulness and ToM. First, mindfulness interventions enhance executive attention, a core component of mentalizing and person understanding [13]. Deployed effectively, executive attention enables perceivers to form multifaceted evaluations and impressions of other social agents that extend beyond rigid stereotype-based conceptions [1]. Second, present-moment thinking facilitates the cognitive operations that map the minds of self and others [14]. Specifically, cortical regions that support mindreading and self-referential mental activity (e.g., medial prefrontal cortex, temporal parietal junction) also play a prominent functional role during mindfulness meditation [2], [15]–[17]. Overlap in these regions likely captures the influence of self-reflection during explicit mindreading [18]. Collectively these observations suggest a straightforward prediction, mindfulness meditation may enhance ToM.

To explore this possibility, participants completed two complementary ToM tasks following a mindfulness (cf. control) intervention. One task, ‘The Reading the Mind in the Eyes Test’ [19], assessed participants’ ability to decode emotions and mental states from subtle facial cues. The other task, ‘The Cyberball Social Exclusion Game’ [20] explored their capacity to empathize with others. Adoption of these tasks enabled us to consider the effects of mindfulness on two pivotal components of ToM, mental state attribution and empathic understanding [3]. Importantly, participants had no prior experience with meditation and did not complete the typical 8-week mindfulness-training program. Instead, only a brief mindfulness-intervention was employed (see also [21]).

## Methods

### Participants and Design

An *a priori* sample size calculation performed on G*Power 3.0.10 (∝ = .05, *d* = 0.6, power = 80%) revealed a requirement of 72 participants. Seventy-two individuals (36 females; *M_age_* = 23.8, range 18–50 years; 93% Chinese) provided written consent and took part in the research. The ethics board at the University of Hong Kong approved the manner of consent and the study (reference EA 480114). The experiment had a single factor (Condition: mindfulness or control) between-participants design. No rewards were offered for participation in the research, all participants received de-briefing and opportunity for further clarification afterwards.

### Materials and Procedure

Participants were greeted by a female experimenter, randomly assigned to one of the treatment conditions (i.e., mindfulness or control), and told the research comprised an investigation (i.e., series of tasks) into people’s reactions to different types of thoughts and situations (i.e., participants were blind to the purpose of the inquiry). The experimental manipulation was then introduced. All participants were instructed to close their eyes, relax and listen to scripted audio instructions (via headphones) and that a bell would chime after 5-minutes to signal the end of this activity. Based on an established protocol [22], [23], participants in the mindfulness condition were instructed to pay particular attention to the sensation of their breathing during the 5-minute period. In addition, they were told it is quite natural for the mind to be distracted and attention to wander during such a task. However, they were asked to observe these moments as fleeting states of mind and to return attention to their breathing each time a distracting thought, emotion or memory occurred. Participants in the control condition received instructions that were similar in style and length. Contrasting the mindfulness treatment, however, these individuals were told to notice each thought, emotion and memory that may arise and to be completely immersed in the experience [21].

Following the 5-minute task, as a manipulation check, participants completed the Mindful Attention Awareness Scale (MAAS-State, [24]), a questionnaire (5-item) that probes levels of mindful-attention and awareness via a 7-point rating scale (0 = not at all; 6 = very much). On completion of the MAAS-S, participants’ mindreading skills and levels of empathic understanding were assessed using the Reading the Mind in the Eyes Test [19] and Cyberball Social Exclusion Game [20], respectively. Task order was counterbalanced across the sample.

### Reading the Mind in the Eyes Test (RMET)

The RMET required participants to identify the emotions/mental states expressed by eyes displaying subtle affective facial expressions. Measuring mental state inference from socio-perceptual cues, the RMET is one of the most widely used instruments in ToM research [25]. In total, participants viewed 36 pairs of eyes (black-and-white pictures of 18 male and 18 female eyes) displaying discrete expressions (12 negative, 8 positive, 16 neutral, counterbalanced for the sex of the eyes). Their task was to report which of four presented words (e.g., hateful, jealous, arrogant, panicked) best described the emotion/mental state of the person whose eyes were displayed on each trial.

### Cyberball Social Exclusion Game

Participants were asked to watch a 90-second video clip during which three players (i.e., cartoon figures) engaged in a virtual ball-tossing game (i.e., Cyberball, cyberball.wikispaces.com). The names of the three female players were provided and participants were informed that afterwards they would have an opportunity to write a letter to Ann, one of players. In total, participants observed two rounds of Cyberball (11 ball tosses per round). Critically, during each round, Ann received the ball on only two occasions, thus was largely ignored by the other players. This paradigm has been used extensively (and successfully) in previous research to simulate social exclusion/rejection and distress (e.g., [26], [27]. Following the Cyberball videos, participants were given 3-minutes to write a letter to Ann, and were instructed to provide an account of their thoughts and feelings during the game. These letters were later scored for empathic content by independent raters. On completion of the tasks, participants were debriefed, thanked and dismissed.

## Results

Several *t*-tests were conducted in order to examine inter-group differences. The success of the experimental manipulation were confirmed, such that participants in the mindfulness condition reported a greater awareness of the present moment (i.e. state mindfulness) than their counterparts in the control condition, *t*(70) = 6.23, *p*<.001, *d* = 1.47 (see Table 1). As expected, participants who underwent the mindfulness induction reported higher levels of state mindfulness.

**Table 1:** Descriptive statistics as a function of experimental condition.

	Condition		
	Mindfulness *M* (*SD*)	Control *M* (*SD*)	95% CI
Measure			
MAAS-S	4.31 (0.98)	2.89 (0.95)	1.87 0.96
RMET	26.42 (3.01)	21.97 (3.67)	6.02 2.87
Empathy	3.50 (1.46)	2.58 (1.50)	1.61 0.22

CI – confidence interval; *M* – mean; MAAS-S - Mindful Attention Awareness Scale-State; RMET - Reading the Mind in the Eyes Test; *SD* – standard deviation.

Mindreading performance, as measured by the RMET, was better for participants in the mindfulness than control condition, *t*(70) = 5.62, *p*<.001, *d* = 1.33, confirming our prediction. Additional regression analyses were undertaken to test the predictive power of mindfulness after controlling for other variables (i.e., sex and age). Using the enter method, a significant model emerged in which mindfulness best predicted mindreading scores (standardized ß = −.41, *p*<.001 and an adjusted variance *R* ^2^ = .13, *p*<.001). Neither age nor sex were significant predictors in this model (ß = .05, *p* = .66; ß = .05, *p* = .65; respectively).

**Figure 1:**
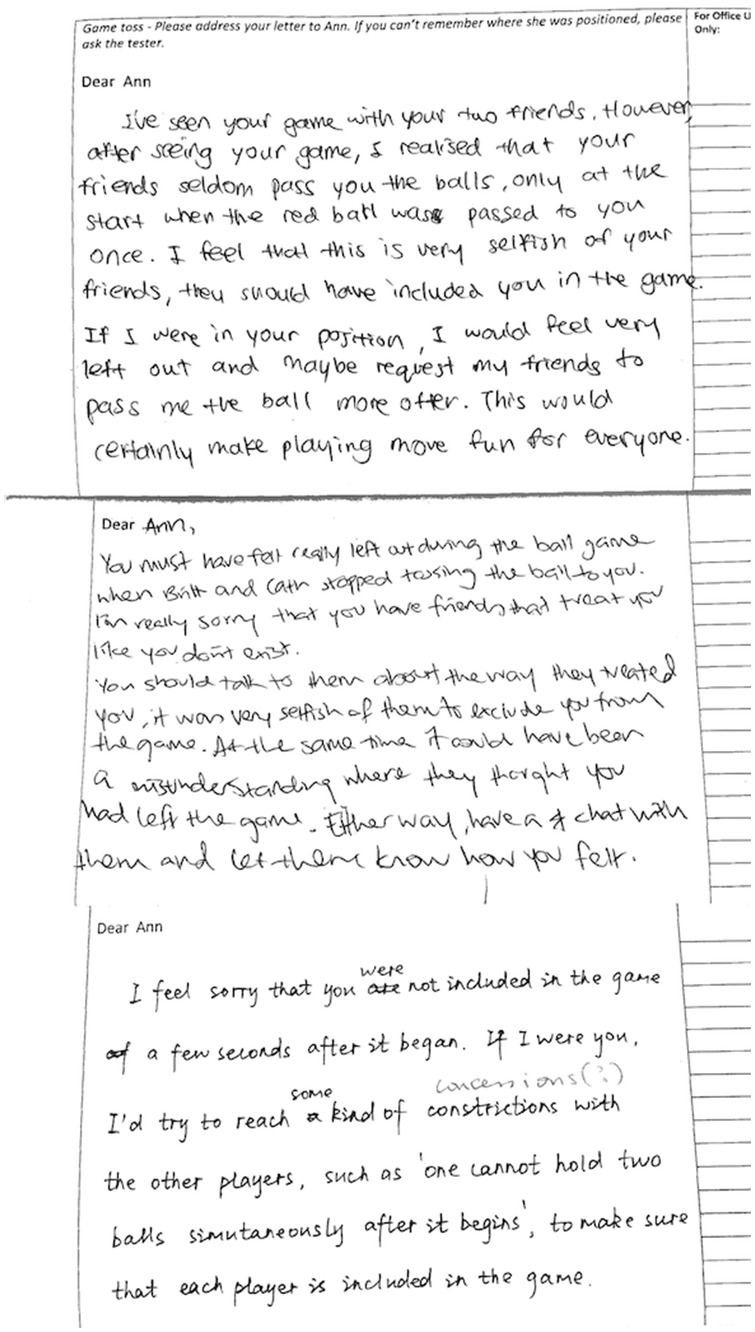
Examples of empathic letters written by participants in the mindfulness condition. These participants expressed more empathic concern, warmth and support in their letters towards Ann (who was socially excluded in the game).

**Figure 2:**
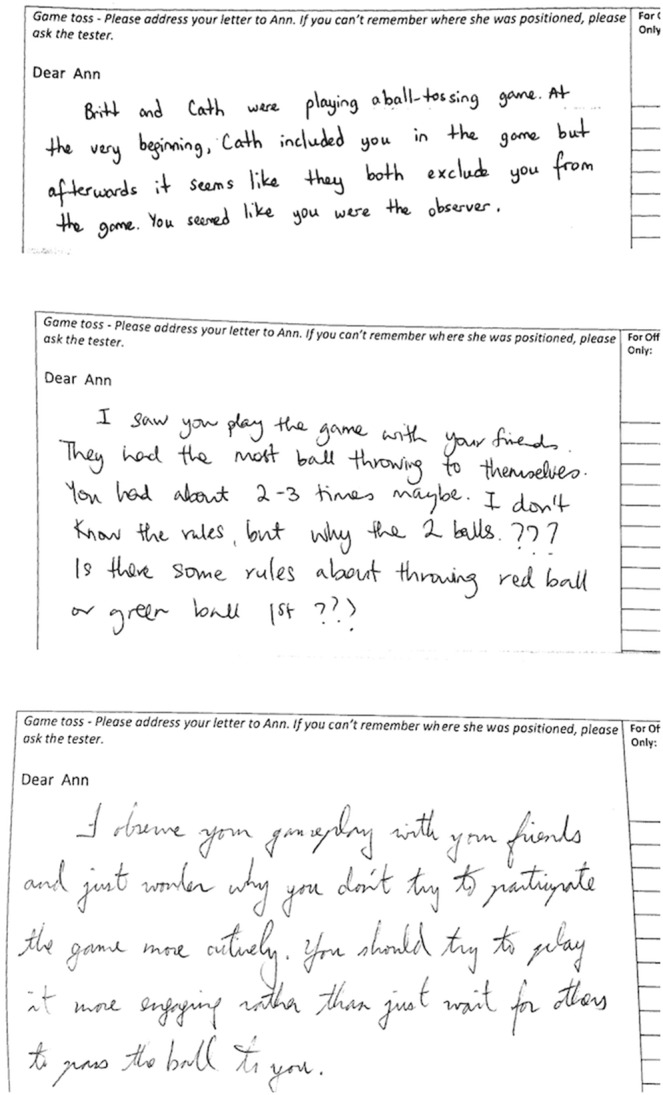
Examples of letters written by participants in the control condition. These participants approached the letter writing task in a *‘matter of fact’* manner; focusing on the facts or rules of the game.

To assess the effect of mindfulness on empathic understanding, two coders who were blind to experimental condition and the purpose of the investigation scored the letters for empathic content (i.e., 6-point scale: 1 = not at all empathic; 6 = very empathic). The coders were instructed to consider the pro-social flavor of the letters, specifically the extent to which the writers had attempted to direct support, comfort and understanding to Ann (i.e., the excluded individual). Assessment of the inter-rater reliability indicated a high level of agreement in their ratings (Cronbach’s *α* = .92), scores were therefore averaged and a single measure of empathic understanding was calculated for each letter (see Table 1). Subsequent between-group analysis revealed that mindfulness participants expressed more empathic concern compared to participants in the control condition, *t*(70) = 2.62, *p* = .011, *d* = .62 (see Figures 1 and 2 for examples of letter excerpts). Further analysis revealed that the sex of participants did not impact empathic concern, *t*(70) = 1.31, *p* = .20.

In summary, significant between-group differences were found after a brief mindfulness induction. Confirming our hypotheses, participants in the mindfulness group out-performed those in control condition on both ToM tasks (i.e. mind reading and empathic expression).

## Discussion

As demonstrated herein, brief mindfulness meditation enhanced core components of ToM, notably mindreading and empathic understanding. Not only were participants better able to decode complex mental states from subtle facial cues following a brief period of mindfulness meditation, so too they expressed greater empathic concern when communicating with the excluded victim of a computerized ball-tossing game. Together, these findings highlight the potent effects that even short-lived mindfulness interventions can exert on basic aspects of social-cognitive functioning (i.e., mentalizing & empathizing).

Questions remain, however, regarding the specific mechanism through which mindfulness meditation impacts ToM? One possibility is that elevated levels of metacognitive awareness facilitate mindreading and boost empathic responding [1]. A commonly articulated viewpoint is that awareness of one’s own bodily and psychological states serves as an important precursor to mentalizing. That is, accurate observations of self are required for a comprehensive understanding of others [18]. As mindfulness meditation focuses on internal (bodily and psychological) experiences, increased self-focused attention is a natural byproduct of this activity [28], [10]. It may therefore be the case that enhanced metacognitive awareness serves as the critical pathway through which brief periods of mindfulness improve ToM [29], a possibility that awaits empirical investigation.

Additional questions center on the extent to which mindfulness interventions benefit all aspects of ToM. In charting people’s mindreading skills, a fundamental distinction has been drawn between the affective and cognitive sub-components of ToM [30]. While affective ToM represents the ability to intuit what a person is feeling, cognitive ToM reflects the capacity to infer their beliefs and intentions. As the current inquiry employed tasks that consider only the affective component of ToM (i.e., RMET, empathy task), it remains unclear whether comparable improvements in mindreading would emerge on activities that tap the cognitive aspects of person understanding (e.g., false belief tasks, see also [7], [9]. Given however that mindfulness is acknowledged to enhance awareness of bodily states, emotions and cognitions [28], there is little reason to suspect that improvements in mindreading should be restricted to affective tasks. Instead, elevated metacognitive awareness may facilitate multiple strands of person understanding [1].

While previous work has highlighted the cognitive and emotional benefits of long-term meditation training [10], here we demonstrated comparable effects on mindreading following only a brief (i.e., 5-minute) mindfulness intervention. The current results are far from unique, however. Elsewhere, researchers have shown that even brief mindfulness training can improve performance in a variety of domains, including: executive function, visual-spatial processing, working memory and impulse control (e.g., [31], [22], [32]). Of course, that brief episodes of mindfulness appear to facilitate processing much like extended training regimes raises a host of important questions pertaining to the underlying processes, mechanisms of change, strength, and duration of the respective effects. In no sense are we suggesting that brief mindfulness interventions are as effective as long-term training programs in shaping behavior. Nevertheless, emerging evidence indicates that even minimal mindfulness training is sufficient to facilitate fundamental aspects of human cognition. Elucidating the precise nature of these effects will be an important task for future research [32].

## Conclusions

The finding that brief mindfulness training improves ToM may have implications that extend beyond the laboratory. Underlying harmonious living is the ability to understand the thoughts and feelings of other social agents. Indeed, ToM is deemed by many to be one of the pinnacles of human evolution. While extant research has focused primarily on explicating the problems that emerge when mindreading goes awry, recent investigations have shifted instead to the identification of strategies (and interventions) that enhance person understanding [8]. As the current findings demonstrate, brief mindfulness-based meditation comprises just such a tactic. At least with respect to affective ToM, mindful attention facilitates mindreading, although additional research will be required to scrutinize the hypothesized link between mindfulness and ToM. Of particular importance will be work exploring the process and consequences of brief mindfulness meditation in applied settings, such as a longitudinal study with a clinical population.
